# Persisting Shadows: Unraveling the Impact of Long COVID-19 on Respiratory, Cardiovascular, and Nervous Systems

**DOI:** 10.3390/idr15060072

**Published:** 2023-12-15

**Authors:** Christina-Michailia Sideratou, Christos Papaneophytou

**Affiliations:** Department of Life Sciences, School of Life and Health Sciences, University of Nicosia, 2417 Nicosia, Cyprus; christin.sideratou@gmail.com

**Keywords:** SARS-CoV-2, COVID-19, long-COVID, post-COVID, cytokine storm, ACE-2

## Abstract

The coronavirus disease 2019 (COVID-19), instigated by the zoonotic Severe Acute Respiratory Syndrome Coronavirus 2 (SARS-CoV-2), rapidly transformed from an outbreak in Wuhan, China, into a widespread global pandemic. A significant post-infection condition, known as ‘long- COVID-19′ (or simply ‘long- COVID’), emerges in a substantial subset of patients, manifesting with a constellation of over 200 reported symptoms that span multiple organ systems. This condition, also known as ‘post-acute sequelae of SARS-CoV-2 infection’ (PASC), presents a perplexing clinical picture with far-reaching implications, often persisting long after the acute phase. While initial research focused on the immediate pulmonary impact of the virus, the recognition of COVID-19 as a multiorgan disruptor has unveiled a gamut of protracted and severe health issues. This review summarizes the primary effects of long COVID on the respiratory, cardiovascular, and nervous systems. It also delves into the mechanisms underlying these impacts and underscores the critical need for a comprehensive understanding of long COVID’s pathogenesis.

## 1. Introduction

Infection with severe acute respiratory syndrome coronavirus-2 (SARS-CoV-2) leads to an acute multisystem illness known as coronavirus disease 2019 (COVID-19) [1]. This infection has resulted in a significant global pandemic with considerable mortality and morbidity [2]. While about 80% of affected individuals experience mild to moderate disease, 5% develop critical illness [3]. The common signs of COVID-19, including shortness of breath, high body temperature, coughing, and tiredness, can lead to serious health issues like lung infection, heart inflammation, and kidney damage [4].

SARS-CoV-2, an airborne zoonotic virus, primarily employs the angiotensin-converting enzyme 2 (ACE2) receptor for cell entry by binding its spike protein to the receptor; however, other receptors might also be involved [5]. ACE2, crucial in COVID-19 pathogenesis, is abundantly found in various tissues, including the lungs, heart, liver, kidneys, gastrointestinal tract [6], and nervous system [7]. As a result, COVID-19 often manifests multi-organ damage, leading to conditions like acute myocardial injury, acute kidney injury, and acute respiratory distress syndrome (ARDS) [8,9].

The genome of SARS-CoV-2 is approximately 79% homologous to severe acute respiratory syndrome 1 (SARS-CoV-1) and 50% homologous to the Middle East Respiratory Syndrome Coronavirus (MERS-CoV) genome [10]. As per the findings from the International Committee on Taxonomy of Viruses (ICTV) and the European Center for Disease Control (ECDC), SARS-CoV-2 is classified within the Coronaviridae family, the Orthocoronavirinae subfamily, and lineage B of the genus Coronaviruses [11]. The viral particles have a diameter ranging from 60 to 140 nm [12]. While the predominant shape of these particles is spherical or ellipsoidal, oval shapes have also been reported. The virus possesses an envelope and a helically symmetrical nucleocapsid [13].

The acute phase of COVID-19 generally lasts up to 4 weeks from the onset of the initial infection [14]. However, in a subset of patients, symptoms may continue beyond this period into a post-acute phase known as ‘long COVID-19’. Interestingly, there are instances where patients experience prolonged symptoms for weeks or even months following the initial infection, regardless of its initial severity [15]. This has captured the attention of numerous organizations and research groups, including the World Health Organization (WHO), National Institute for Health and Care Excellence (NICE), National Health Service (NHS), and Centers for Disease Control and Prevention (CDC). This lingering condition has received various names, including ‘post-acute sequelae of SARS-CoV-2 infection’, ‘post-acute COVID-19 syndrome’, ‘long-COVID-19’, ‘long-COVID,’ ‘long haulers COVID-19’, ‘long haulers,’ and ‘post-COVID syndrome’. Post-SARS-CoV-2 implications pose a public health challenge with potentially severe repercussions [16]. However, definitions vary among authorities, particularly concerning the duration of symptoms that are classified as “long-haul”. According to the CDC (https://www.cdc.gov/coronavirus/2019-ncov/long-term-effects/index.html, accessed on 15 September 2023), long COVID is a “Wide range of new, returning, or ongoing health problems people can experience four or more weeks after first being infected with the virus that causes COVID-19”. However, the WHO defined long COVID as an “Illness that occurs in people who have a history of probable or confirmed SARS-CoV-2 infection, usually within three months from the onset of COVID-19, with symptoms and effects that last for at least two months, that cannot be explained by an alternative diagnosis” (https://www.who.int/news-room/questions-and-answers/item/coronavirus-disease-(covid-19)-post-covid-19-condition, accessed on 15 September 2023). According to the definition proposed by the NHS, long COVID is observed when “Symptoms lasting weeks or months after the infection has gone” (https://www.nhs.uk/conditions/covid-19/long-term-effects-of-covid-19-long-covid/, assessed on 15 September 2023). On the other hand, two different definitions for long COVID have been proposed by the NICE Institute for Health and Care Excellence [17]: (i) “Ongoing symptomatic COVID-19 for people who still have symptoms between 4 and 12 weeks after the start of acute symptoms”; and (ii) “post-COVID-19 syndrome for people who still have symptoms for more than 12 weeks after the start of acute symptoms”.

The variability mentioned above in definitions for long COVID-19 complicates the establishment of a unified criterion for research. To address this, the WHO recently defined long COVID-19 (Post COVID-19) as a condition appearing in individuals with a history of suspected or confirmed SARS-CoV-2 infection, typically three months post-infection, with symptoms persisting for at least two months and not explained by an alternative diagnosis (reviewed in [18]).

The scientific community has been actively conducting research since the first reported case of COVID-19, caused by the SARS-CoV-2, in early December 2019 in China [19]. With 771,820,937 cases and 6,978,175 deaths reported thus far, evidence suggests that symptoms can persist long after the acute phase of the infection (https://covid19.who.int, accessed on 1 November 2023).

This review aims to summarize the symptoms encountered in the long COVID-19 period in the respiratory, cardiovascular, and nervous systems in particular, as well as to evaluate the possible mechanisms underlying these symptoms and explain them. We searched PubMed (Medline), ScienceDirect, WHO, and CDC websites using keywords related to long-term COVID-19 effects, SARS-CoV-2, and the pandemic. Keywords such as “long COVID-19”, “post COVID-19 syndrome”, and “pandemic” were combined with Boolean operators for optimal retrieval. The search spanned 1 January 2020, to 31 August 2023. Eligible studies were original research articles in English on SARS-CoV-2 and its long-term effects. We included studies of all designs, offering insights into the listed categories. We excluded studies on pediatric populations and those with follow-up periods shorter than 25 days post-acute phase.

## 2. Health Implications Related to Long COVID

COVID-19, caused by SARs-CoV-2, has systemic implications that extend beyond the respiratory system, profoundly impacting cardiovascular health and other organ systems [20]. This multifaceted virus manifests with a wide array of symptoms affecting multiple organs, ranging from ‘silent hypoxia’—characterized by low blood oxygen levels without breathlessness—to neurological symptoms such as delirium, ‘brain fog’, mood changes, and the unexpected onset of conditions like hypertension or diabetes [21,22,23]. Recent findings suggest that lung inflammation during COVID-19 infection may lead to elevated cortisol levels, a physiological response that can subsequently increase blood pressure and potentially result in vessel injury [24]. Additionally, a critical aspect of SARS-CoV-2’s pathology is its impact on microcirculation. The virus causes endothelial cell swelling and damage (endotheliitis), leading to the formation of microscopic blood clots (microthrombosis), capillary congestion, and damage to pericytes, which play an essential role in capillary integrity, tissue repair, and scar formation. These microvascular changes are integral to understanding the widespread effects of the virus, elucidating the increased risk of vessel injury and subsequent formation of microclots (reviewed in [22]).

Furthermore, numerous biomedical discoveries have been made, with many patients reporting a variety of persisting symptoms following SARS-CoV-2 infection, which are described as “long COVID” affecting multiple organs [25]. The term ‘long COVID’ covers a range of complications, such as cardiovascular, thrombotic, and cerebrovascular diseases [26], type 2 diabetes [26], myalgic encephalomyelitis/chronic fatigue syndrome (ME/CFS) [27], and dysautonomia, notably postural orthostatic tachycardia syndrome (POTS) [28]. These symptoms can persist for extended periods, and conditions like new-onset ME/CFS and dysautonomia are often considered permanent [29]. A significant number of long COVID patients have found it challenging to rejoin the workforce [25], leading to a notable contribution to workforce deficits. As of the time of publication of this work, no treatments have been definitively confirmed as effective. Research into immune irregularities in long-term COVID patients who initially experienced mild COVID-19 symptoms reveals T cell anomalies such as T cell exhaustion, diminished CD4^+^ and CD8^+^ effector memory cells, and increased PD1 expression on central memory cells lasting at least 13 months [30]. A surge in cytotoxic T cells has been linked to the gastrointestinal symptoms of long COVID [31]. Further research indicates elevated cytokine levels, especially IL-1β, IL-6, TNF, and IP10 [32]. A recent study also highlighted prolonged high levels of CCL11 linked with cognitive issues [33].

In the following paragraphs, we explore the impacts of long-term COVID on various bodily systems, specifically the respiratory, cardiovascular (CV), and nervous systems. Additionally, we strive to elucidate the potential mechanisms through which infection with SARS-CoV-2 can result in persistent symptoms and influence these particular systems.

### 2.1. Effect of Long COVID on the Respiratory System

The respiratory system is notably the most commonly affected by SARS-CoV-2. However, respiratory symptoms may persist beyond the acute phase of infection into what is known as the ‘long COVID-19’ phase, even after patients have ostensibly recovered. Various studies have documented abnormalities in pulmonary function tests (PFTs) and chest CT images persisting for months following hospital discharge. Dyspnea and cough have been the most frequently reported persistent respiratory symptoms [34,35,36]. Table 1 summarizes the main findings of studies focusing on the effects of long COVID on the respiratory system.

Schwensen et al. [41] suggested that pulmonary fibrosis (PF) may be a long-term complication in patients who have experienced severe COVID-19. They reported a case involving an 80-year-old patient with no prior history of lung disease whose lung CT scan was normal two months prior to SARS-CoV-2 infection. However, a high-resolution CT scan on day 39 post-infection revealed bilateral consolidations, septal thickening, traction bronchiectasis, and infiltrative and parenchymal changes indicative of extensive pulmonary fibrosis. The comparative analysis of CT scans demonstrated the development of significant fibrosis in previously healthy lungs. Consequently, the study highlights that acute respiratory distress syndrome (ARDS), which was reported in up to 42% of hospitalized COVID-19 patients, could be a contributing factor to the development of pulmonary fibrosis.

It has been previously demonstrated that ACE2 is linked to acute lung injury. One proposed mechanism as far as fibrosis development resulting from the previous SARS pandemic is the direct stimulation of the Transforming Growth Factor-β (TGF-β) by the nucleocapsid protein of SARS-CoV-1. Since the nucleocapsid core of SARS-CoV-2 is almost 90% similar to that of SARS-CoV-1, it may be valid [42]. The downregulation of ACE, which further regulates Angiotensin II, may lead to the stimulation of TGF-β. In addition to TGF-β, the production of advantageous factors such as Platelet-Derived Growth Factor (PDGF) and Vascular Endothelial Growth Factor (VEGF) is also found, resulting in the activation of fibroblasts, which are the activated macrophages and neutrophils that release pro-fibrotic mediators that promote the accumulation of myofibroblasts by stimulating collagen production [43].

Pulmonary myofibroblasts can arise from various progenitors, and they typically undergo apoptosis, concluding the healing process [44]. Following their differentiation from fibroblasts, myofibroblasts stimulate collagen synthesis. However, during fibrosis, the normal cessation of extracellular matrix (ECM) production is disrupted, and increased tissue stiffness exacerbates cell injury, leading to further myofibroblast activation [45]. This creates a self-sustaining loop of activation, resulting in irreversible fibrotic changes. These cells organize into fibrotic foci within the lung tissue [46]. Growth factors, particularly those targeting tyrosine kinase pathways, persistently stimulate the formation and development of these fibrotic areas, which may regress naturally or progress to chronic pulmonary fibrosis due to excessive collagen buildup [47]. During a SARS-CoV-2 infection, the virus targets respiratory epithelial cells, triggering local innate immune responses that include the release of inflammatory cytokines and chemokines. These inflammatory mediators recruit additional immune cells such as monocytes, macrophages, neutrophils, dendritic cells (DCs), and natural killer (NK) cells and activate adaptive immune responses involving CD4^+^ and CD8^+^ T cells. The continued presence of inflammatory cytokines like IL-2, IFN-γ, and TNF-α promotes myelination and urgent granulation tissue formation, aggravating lung injury and epithelial damage [48]. These cytokines increase lung capillary permeability, leading to diffuse bilateral ground-glass opacities, hypoxemia, and, ultimately, long-term fibrotic alterations [49]. The cytokine storm induced by SARS-CoV-2 infection can result in severe respiratory complications such as ARDS. Lung autopsies from COVID-19 fatalities have shown significant macrophage infiltration in the bronchial mucosa, confirming the extent of the inflammatory response [50].

As aforementioned, the lung is the primary target organ of SARS-CoV-2 infection [51]. In the intricate landscape of COVID-19’s impact on the respiratory system, a critical distinction emerges between upper airway inflammation, typically associated with milder cases and quicker recovery, and alveolar inflammation, often indicative of more severe infections and a precursor to extensive lung fibrosis [52]. Pandolfi et al. [53] examined the correlation of broncho-alveolar inflammation in COVID-19 patients with disease severity. Their study analyzed 33 adults admitted to either the intensive care unit (ICU) or the intermediate medicine ward (IMW) using bronchoalveolar lavage (BAL). Results indicated higher neutrophil counts and lower lymphocyte and macrophage levels in ICU patients compared to IMW patients, with elevated levels of pro-inflammatory cytokines IL6 and IL8 in non-survivors. Interestingly, IL10 levels showed no significant variation between groups. Treatment with steroids resulted in lower BAL concentrations of IL6 compared to tocilizumab or antivirals, suggesting that innate immune responses primarily drive alveolitis in COVID-19.

Barilli et al. [54] demonstrated that exposure of alveolar A549 cells to supernatants from S1 spike-activated macrophages significantly increased the release of inflammatory mediators, primarily IL-8. This indicates the involvement of the NF-kB pathway in IP-10 and RANTES transcription, with STATs regulating most cytokines/chemokines. The cytokines/chemokines from activated macrophages disrupted the barrier integrity of Human Alveolar Epithelial Lentivirus-immortalized cells (hAELVi), evident through increased permeability and disorganized claudin-7. This suggests that A549 cells contribute to lung inflammation and alveolar damage, which is crucial in ARDS pathology in COVID-19. In another study, Lazar et al. [55] compared 100 patients with severe pneumonia to a control group of 61 non-COVID patients, finding that 69% of COVID-19 patients showed persistent interstitial changes indicative of fibrotic alterations. The risk of developing fibrosis correlated with higher levels of ESR, CRP, LDH, and length of hospital stay. Imaging analysis revealed an increased risk of interstitial fibrosis with a more significant number of affected pulmonary lobes and a higher percentage of interstitial pulmonary fibrosis. These results indicate that the main risk factors for post-COVID-19 pulmonary fibrosis include elevated ESR, CRP, LDH, prolonged hospitalization, and the severity of the initial pneumonia. Furthermore, pulmonary fibrosis is a recognized sequela of ARDS [56]. While the majority of patients with COVID-19 pneumonia survive the acute phase, the timing for diagnosing irreversible pulmonary fibrosis post-COVID-19 remains unclear [57]. It is still uncertain whether survivors of severe COVID-19 will develop long-term lung complications or whether COVID-19-related pulmonary fibrosis will resolve, persist long-term, or become progressive as observed in human Idiopathic Pulmonary Fibrosis (IPF) [58].

### 2.2. Effect of Long COVID on the Cardiovascular System

SARS-CoV-2 infection impacts the CV system during the acute phase [59]; however, cardiac complications can persist even after recovery from the acute phase of the infection [59,60]. Given the high prevalence of such complications during this stage, it is crucial to pay attention to the potential long-term cardiac implications of the disease. Emerging evidence demonstrates a significant burden on the CV system during the long COVID period (reviewed in [61]). Symptoms specific to the CV system involvement of long COVID include palpitations, chest pain, breathlessness, and postural dizziness with or without syncope [62]. Palpitations and chest pain are the most common findings of the long COVID period [63]; seemingly healthy individuals may experience dizziness and an increased heart rate while resting [64].

Interestingly, in 2020, when long COVID was not yet widely recognized, Puntmann et al. [65] stressed the importance of ongoing monitoring for the long-term CV impacts of COVID-19. They found that among 100 individuals who had recovered from COVID-19, 78 showed abnormal results in cardiovascular magnetic resonance (CMR) imaging. These abnormalities included elevated myocardial native T1 (found in 73 participants), increased myocardial native T2 (in 60 participants), myocardial late gadolinium enhancement (in 32 participants), and pericardial enhancement (in 22 participants). Furthermore, ongoing myocardial inflammation was noted in 60% of the participants, irrespective of their preexisting health conditions, the severity and progress of the acute phase of their illness, or the time since their initial COVID-19 diagnosis. Subsequent studies, like the one by Huang et al. [66] with 26 recovered COVID-19 patients, also found significant cardiac involvement. In this cohort, 58% (15 patients) exhibited abnormal CMR results: 54% (14 patients) showed myocardial edema, and 31% (8 patients) had late gadolinium enhancement (LGE). Patients with abnormal CMR had diminished right ventricular function, including lower ejection fraction, cardiac index, and stroke volume relative to body surface area. These findings suggest cardiac issues, including myocardial edema, fibrosis, and right ventricular dysfunction, are prevalent in some COVID-19 recoverees. Table 2 summarizes the main findings of studies focusing on the effects of long COVID on the CV system.

In another study, Mandal et al. [67] analyzed 384 patients 54 days post-hospital discharge from COVID-19, finding significant ongoing symptoms and health issues. Persistent breathlessness was reported by 53%, cough by 34%, and fatigue by 69%. Additionally, 14.6% exhibited signs of depression. Among those discharged with high biomarkers, 30.1% still had elevated d-dimer levels, and 9.5% had high C-reactive protein levels. Chest X-rays remained abnormal in 38% of the patients, with 9% showing worsening conditions.

Kotecha et al. [68] studied 148 severe COVID-19 patients (32% with elevated troponin) undergoing convalescent CMR about 68 days post-hospitalization. Normal LV function was seen in 89%, but 54% had LGE and/or ischemia. Myocarditis-like scar was noted in 26%, infarction/ischemia in 22%, and both in 6%. The majority of myocarditis-like injuries were minor, affecting a few myocardial segments without impacting LV function, and 30% showed active myocarditis. Myocardial infarction occurred in 19%, and inducible ischemia in 26% of those tested. Notably, 66% with ischemic injury lacked a prior coronary disease history. There was no widespread myocardial edema or fibrosis. The study highlights myocarditis-like injury and localized inflammation in many post-COVID-19 cases, with some showing ischemic heart disease, often without prior history.

In a subsequent study by Puntmann and coworkers [69], among 346 COVID-19 patients without prior cardiac disease, 73% reported cardiac symptoms like dyspnea, palpitations, and chest pain, initially assessed at a median of 109 days after infection. Symptomatic patients exhibited higher heart rates and signs of cardiac inflammation, though severe heart disease or elevated cardiac biomarkers were rare. At a follow-up approximately 329 days post-infection, over half (57%) still experienced cardiac symptoms, with persistent symptoms more common in females and those with initial myocardial involvement. This suggests that ongoing cardiac inflammation may contribute to long-term cardiac issues in previously healthy individuals with mild COVID-19.

In a retrospective study by Dini et al. [39], 180 COVID-19 patients exhibiting persistent or new symptoms ≥12 weeks post-negative SARS-CoV-2 test were examined for potential heart involvement. Following a thorough physical examination, patients with suspected heart issues underwent comprehensive cardiovascular evaluations, including echocardiography, as needed. Among them, 52% reported shortness of breath or fatigue, 34% had chest pain or discomfort, and 37% experienced heart palpitations or arrhythmias. Acute pericarditis was diagnosed in 22% (39 patients), with mild-to-moderate pericardial effusion in some and thickened pericardial layers with small effusions in others. The study found a significant association between heart palpitations/arrhythmias, autoimmune or allergic disorders, and acute pericarditis. It also noted a less likely hospitalization during the initial COVID-19 infection as a borderline contributing factor. The findings highlight a high prevalence of acute pericarditis in long COVID-19 patients, with specific symptoms and conditions linked to increased risk of pericardial disease.

Although the exact pathophysiological connection between long COVID-19 and CV system issues remains inconclusive, conditions like myocarditis and pericarditis may play a role. The studies mentioned above have uncovered a surprisingly high frequency of imaging abnormalities, suggesting widespread myocardial damage and inflammation in these patients. This finding is crucial for comprehending and managing the cardiac symptoms that persist in the extended recovery phase of COVID-19 [64].

The myocardium maintains a critical balance between the classical and non-classical pathways of the renin–angiotensin–aldosterone system (RAAS). An upsurge in the activity of the classical RAAS pathway, coupled with a suppression of the non-classical pathway, is correlated with adverse cardiovascular outcomes [70]. The enzyme ACE2 plays a crucial role in cardiac physiology and pathology. Specifically, the binding of SARS-CoV-2 to the ACE2 receptors on myocardial and endothelial cells results in diminished ACE2 activity, thereby impairing the conversion of angiotensin II (Ang II) to angiotensin-(1-7) [Ang-(1–7)] [70]. This reduction in ACE2-mediated conversion exacerbates the effects of the classical RAAS pathway, which are mediated by Ang II, leading to harmful cardiovascular effects [71].

The heightened activity of Ang II, which is characteristic of the classical RAAS pathway dominance, is associated with a decrease in collagenase activity within the cardiac tissue. This enzyme reduction can contribute to pathological remodeling of both atrial and ventricular myocardium, potentially resulting in detrimental structural and functional changes to the heart [72].

Enhanced binding of angiotensin II (Ang II) to the Ang II Type 1 Receptor (AT1R) initiates a signaling cascade that leads to phosphorylation and increased catalytic activity of a Disintegrin and Metalloproteinase 17’ (ADAM-17). Activation of ADAM-17 promotes the shedding of ACE2 from the cell surface, further decreasing Ang II clearance. The result is an amplification of Ang II-induced inflammatory responses, creating a self-perpetuating cycle of inflammation [72]. Moreover, the reduction in ACE2 activity can contribute to myocardial fibrosis, potentially leading to symptoms such as fatigue and dyspnea, characteristic of post-acute sequelae of SARS-CoV-2 infection [25].

Myocardial injury in COVID-19 may result from indirect effects mediated by the systemic inflammatory response [73], primarily through the “cytokine storm” phenomenon [74]. In the context of a SARS-CoV-2 infection, cytokine storms can activate bone marrow-derived endothelial cells, resulting in pericardial inflammation [75]. The adverse inotropic effects of pro-inflammatory cytokines can impair cardiac contractility. Persistent activation of inflammatory signaling, mainly via tumor necrosis factor-alpha (TNFα) and interleukin-1 beta (IL-1β), can lead to widespread cardiomyocyte apoptosis and subsequent abnormal left ventricular remodeling, predisposing to acute heart failure. Furthermore, cytokine storms stimulate monocytes/macrophages to release matrix metalloproteinases, accelerating the growth and rupture of atherosclerotic plaques, promoting the release of procoagulant factors, and causing hemodynamic changes, thus elevating the risk of Acute Myocardial Infarction (AMI) [76].

Cytokine storms are also linked with lymphopenia, characterized by reduced lymphocyte counts [77]—the inflammatory response results in lymphocyte depletion, impairing the body’s ability to fight the SARS-CoV-2 infection. Consequently, cytokine production is deregulated, leading to damage to healthy cells, initially in the lungs and potentially extending to other organs, including the heart [78].

### 2.3. Effect of Long COVID on the Nervous System

Individuals with long COVID can exhibit a broad spectrum of symptoms, including persistent neuropsychiatric issues that may arise or persist for months following the initial infection [25,79,80]. Recognized as a condition affecting multiple organs, long-term COVID-19 definitively involves both the Central Nervous System (CNS) and Peripheral Nervous System (PNS), contributing to the enduring nature of the disease [81,82]. Approximately one-third of individuals who test positive for SARS-CoV-2 experience neurological and neuropsychiatric symptoms early in the course of the disease, and these symptoms can persist long after the acute infection has resolved. Commonly reported symptoms include anosmia (loss of smell), ageusia or dysgeusia (altered taste), headache, fatigue, cognitive impairment (‘mental fog’), and memory loss, enduring for weeks or even months [83,84]. Other observed issues include impaired concentration, sensory disturbances, and depression [85]. Numerous studies conducted globally have consistently reported fatigue as the most frequent and debilitating symptom of long COVID-19, independent of the disease’s initial severity or the occurrence of respiratory distress [86,87]. Moreover, SARS-CoV-2 infection can precipitate inflammatory neurological syndromes, such as encephalitis and acute disseminated encephalomyelitis, along with ischemic and hemorrhagic strokes [88]. Table 3 summarizes the main findings of studies focusing on the effects of long COVID on the central nervous system.

SARS-CoV-2 has brain tropism, and the neurological dysfunctions that have been reported may be due to the Renin–Angiotensin System (RAS) damage of the nervous system. The imbalance of the two aspects of RAS: (1) ACE/Ang II/AT1R, and (2) ACE2/Ang-(1–7)/Angiotensin II Type 2 Receptor (AT2R) in the brain leads to neuroinflammation, neurotoxicity, and Blood–Brain Barrier (BBB) disruption among other things. AT1R, among others, causes inflammation and oxidative stress [93]. AT2R has an essential role in neuraxon regeneration, i.e., it protects the brain by conducting neuronal survival, and in the case of SARS-CoV2 infection, it protects one against the deleterious effects of AT1R along with MasR [94].

Various post-COVID-19 effects, such as hyposmia/anosmia and memory/cognitive impairment, have been attributed to hypometabolism in different areas of the brain. For example, hypometabolism of the brainstem is associated with hyposmia/anosmia, while hypometabolism of the cerebellum or frontal cortex is linked to memory/cognitive impairment. The Positron Emission Tomography (PET) scans of long COVID patients who express persistent complaints at least three weeks after the onset of their acute infection symptoms showed hypometabolism in their bilateral rectal/orbital helix (containing the olfactory helix) in the right temporal lobe (amygdala and hippocampus extending into the right thalamus), in the bilateral brainstem bridge/myelin and the bilateral cerebellum. These findings could indicate the involvement of the ACE2 receptor in the neurotropism of SARS-CoV-2, particularly in the olfactory bulb. This is likely due to the dissemination route from the nose to the olfactory bulb, where the ACE2 receptor has a strong presence; it has been hypothesized that the ACE2 receptor is the cause of several coronaviruses [95].

As aforementioned, cytokine storm, a systemic hyperinflammatory state characteristic of the acute phase of COVID-19, activates neuroglial cells and increases the risk of neurological complications post-infection [96]. Various viruses, including SARS-CoV-2, can infiltrate the CNS through hematogenous routes, triggering immune-induced neurological disorders [97]. SARS-CoV-2 has neurotropic properties; during severe infections, it can infect brain-resident cells such as macrophages, microglia, and astrocytes. These cells, when infected, contribute to a pro-inflammatory state within the CNS [98].

The cytokine storm can also induce cerebral perfusion anomalies, compromise the integrity of the BBB, disrupt astrocytic functions essential for synaptogenesis, and cause neurotransmitter imbalances [99]. This cascade of events can dysregulate neurogenesis and disrupt the normal functioning of neurons, oligodendrocytes, and neuroglial cells [100]. Consequently, disturbances in neuronal plasticity, synaptic function, myelination, and BBB maintenance may lead to cognitive deficits and an array of long-term neuropsychiatric symptoms associated with COVID-19 [101]. Elevated pro-inflammatory cytokine levels have been implicated in causing confusion and altered consciousness [102], and the excessive release of cytokines and chemokines can also result in brain damage through microglial activation [103]. Additionally, an imbalance between TH17 cells and regulatory T cells (Tregs) has been linked to learning and sleep disturbances [104].

Several studies, including cohort studies, have also linked COVID-19 to the development of peripheral nervous system (PNS) diseases, indicating that SARS-CoV-2 infection leads to immune dysregulation affecting the PNS as well [105,106]. Manifestations like nerve pain, skeletal muscle injury, Guillain-Barré syndrome (GBS), cranial polyneuritis, neuromuscular junction disorders, neuro-ophthalmological disorders, neurosensory hearing loss, and dysautonomia have been reported in COVID-19 patients [107]. For instance, Kaeley et al. [108] described a case of a 40-year-old woman with COVID-19 presenting acute GBS, which was not preceded by other infections. Additionally, studies by Filosto et al. [109] and Fragiel et al. [110] reported an increased incidence and severity of GBS during COVID-19 outbreaks in Italy and Spain, respectively.

In a case series examining psoas muscle and femoral nerve biopsies from 35 deceased individuals, 24 patients showed signs of inflammatory/immune-mediated myopathy [111]. The exact link between COVID-19 and GBS is still under investigation. GBS, an inflammatory disorder, is thought to be caused by a host response to infections leading to nerve and nerve root damage. COVID-19 provokes a cytokine immune response, activating Th1 cells and CD14^+^ and CD16^+^ monocytes, resulting in a cytokine storm characterized by elevated IL-6, TNF-α, and other cytokine levels.

Ocular muscle abnormalities, such as pain with eye movements, extraocular movement abnormalities, Adie’s pupil, diplopia, and strabismus, have been observed in both elderly and young COVID-19 patients [112]. In a case study involving five COVID-19 patients with neurological symptoms, neurological examinations revealed flaccid paresis with limb predominance and unilateral facial nerve involvement [113]. Peripheral neuropathy symptoms in COVID-19 patients, such as sudden numbness, limb pain, and weakness, have been noted, along with debates against Guillain-Barre due to the sudden onset of symptoms, lack of ascending pattern, and normal cerebrospinal fluid (CSF) [114].

Furthermore, Oaklander et al. [115] evaluated peripheral neuropathy in patients with prolonged long COVID symptoms. Their study of 17 patients without prior neuropathy history revealed significant neuropathy evidence, with 63% of skin biopsies, 17% of electrodiagnostic tests, and 50% of autonomic function tests confirming the diagnosis. Common diagnoses included small-fiber neuropathy, critical illness axonal neuropathy, and multifocal demyelinating neuropathy, typically occurring within one month of mild COVID-19 infection. Despite an average improvement of 52%, none reported complete symptom resolution, with 65% receiving immunotherapies. These findings suggest small-fiber neuropathy is a common outcome of long COVID, potentially due to immune dysregulation caused by the infection.

In more than one-third of patients with a history of severe SARS-CoV-2 infection, involvement of the central or peripheral nervous system has been observed, with a higher incidence of neurological symptoms reported in patient studies [81]. The most frequent long-COVID neurological manifestations include fatigue, ‘brain fog’, headache, cognitive impairment, sleep, mood, smell or taste disorders, myalgias, sensorimotor deficits, and dysautonomia. Current understanding of the pathophysiological mechanisms involved in long-COVID is limited, but neuroinflammatory and oxidative stress processes are believed to play a significant role in propagating these neurological sequelae [81].

## 3. Potential Mechanisms Resulting in Long COVID-19

The long-term COVID-19 syndrome is still poorly understood by the scientific community, although it affects a relatively large proportion of acute COVID-19 survivors. This work highlights the impact of long-term COVID-19 on the cardiac, nervous, and respiratory systems. Consequently, the pathophysiological mechanisms proposed for persistent symptoms involve:

(i) Direct Damage of the organs/system via the ACE2 receptor: ACE2 has a pivotal role in developing cardiac, brain, and pulmonary complications, as already mentioned [43,93,116]. The Renin–Angiotensin–Aldosterone System (RAAS) is a signaling pathway that acts as a homeostatic regulator of vascular function. Τhe Ang II depending on the receptor type, can have different effects: the classic effects (increased oxidative stress, inflammation, fibrosis, and vasoconstriction) and the opposite effects [117]. The ACE2 is a homolog of the ACE [118] and has a vital role in the RAAS. ACE2 regulates the action of ACE by decreasing the amount of Ang II and increasing the amount of Ang-(1–7). Furthermore, the ACE2, beyond participating in the RASS system, is the gateway for the entry of SARS-CoV-2 [77]. The virus competes with Ang II, and its binding blocks the ACE2 activity. This decreases the enzyme activity at the membrane, resulting in an imbalance of ACE/ACE2 and, consequently, the RASS. This imbalance will lead to an increase in the abnormal activation of the ACE/Ang II/AT1 receptor axis and thus an increase in the Ang II vasoconstriction and a decrease in the Ang vasodilation (1-7) [119].
(ii) Indirect damage through the immune system: Myocardial injury in COVID-19 can occur indirectly through an overactive inflammatory response, often referred to as a ‘cytokine storm’ [73,74]. This hyperinflammatory state poses a significant risk not only to the cardiovascular system but also to the brain and respiratory tissues [48]. Typically, COVID-19 patients exhibit an imbalanced immune profile: an overzealous innate immune response coupled with a diminished adaptive immune response. This manifests as a reduction in various immune cells—lymphocytes, cytotoxic and helper T cells, B cells, and NK cells—particularly in severe cases [120]. The cytokine storm triggered by SARS-CoV-2 infection results in the rampant release of proinflammatory cytokines, creating a disequilibrium between proinflammatory and anti-inflammatory processes [49,121,122]. Elevated levels of interleukin-6 (IL-6) during the acute phase have prompted investigations into its role in long-term COVID-19 sequelae [123,124,125], suggesting that persistent inflammation could underlie the pathophysiology of Long COVID. Further research is essential to elucidate these mechanisms and identify effective treatments to improve the long-term outlook for patients.
(iii) Therapeutic implications: Antiretroviral therapies, including azithromycin and tocilizumab, have been associated with electrophysiological alterations and potential interactions with cardiovascular drugs, warranting cautious use and monitoring [74]. Concurrently, the role of RAAS inhibitors in modifying ACE2 levels is under scrutiny, given their potential dual impact on the disease process [126]. Moreover, severe COVID-19 cases requiring prolonged mechanical ventilation can suffer from heightened intrapulmonary pressure, leading to or exacerbating pulmonary fibrosis [127]. Additionally, the high concentrations of oxygen used to treat critically ill patients can generate free radicals, damaging pulmonary epithelium and contributing to oxidative stress. This stress not only perpetuates the inflammatory state but also may activate fibrogenic pathways, further complicating recovery [58].
(iv) Sociopsychological factors: The pervasive impact of COVID-19 extends beyond the physical to the psychological, with social isolation, the stress of a novel and potentially fatal virus, and the anxiety surrounding transmission and stigma all contributing to long-term psychiatric conditions. Post-acute sequelae may include PTSD, depression, anxiety, and obsessive–compulsive symptoms [82,128]. The enforced solitude, disruption of normal work routines, and financial strains—compounded by the overarching threat of a global health emergency—can engender loneliness, anxiety, and significant behavioral shifts [129,130]. Consequently, the occurrence of anxiety disorders, depressive states, and cognitive deficits is thought to be multifaceted in origin, encompassing a spectrum of physical, functional, and sociopsychological contributors [92].

Moreover, a possible mechanism that SARS-CoV-2 infection leads to implications in the cardiovascular [76] and CNS systems [131] is the impairment of oxygen transfer and the persistence of vessel injury. COVID-19 is highly aggressive and is accompanied by hypoxia, abnormal clotting, and severe inflammation, so most CNS symptoms are identified as manifestations of peripheral pathologies [132]. Notably, after the massive inflammation, there is the exhaustion of CD4^+^ T cells. During COVID-19 infection, CD4^+^ T-cell activity increases over time due to the number of CD4^+^ T cells specific to SARS-CoV-2 elevating within the days of the emergence of clinical manifestations. In individuals with severe COVID-19 infection, the incidence of SARS-CoV-2-specific CD4^+^ T cells was much lower, which implies that managing COVID-19 illness requires a strong response of CD4^+^ T-cell in the patients infected with COVID-19, the number of SARS-CoV-2-specific CD4^+^ T cells increase with age. Still, their function shifts toward Interleukin-2 (IL-2) production rather than IFN production [133]. However, there is evidence COVID-19 can lead to significant reductions in T cell numbers, particularly in patients requiring intensive care. The surviving T cells in these patients often exhibit signs of functional exhaustion, characterized by higher levels of exhaustion markers such as PD-1. This T cell exhaustion, coupled with the decrease in T cell numbers, may contribute to the persistence of inflammation in COVID-19 patients, which can have further implications for cardiovascular and CNS [134]. Moreover, the increased expression of PD1 on CD8^+^ T cells in patients in ICUs, compared with those not in intensive care and healthy controls, suggests a progression of disease severity in COVID-19 patients. As the severity of the disease increases, there is a concomitant rise in inflammatory cytokine levels, which may drive the depletion and exhaustion of T cell populations [135]. Together, the above suggests that the exhaustion of CD4^+^ T cells, a critical element in the transition from inflammation to repair, likely contributes to the persistence of inflammation and subsequent complications observed in severe COVID-19 cases. This suggests that the observed effects in the cardiovascular system and CNS may be more related to the body’s inflammatory response rather than direct viral invasion of these systems.

While the exact mechanisms leading to long COVID remain inconclusive, research, including a study by Wong et al. [136], suggests that PASC is associated with a reduction in serotonin levels. This study outlines that viral infection, coupled with type I interferon-driven inflammation, can decrease serotonin via three pathways: reduced absorption of tryptophan in the intestines, platelet hyperactivation affecting serotonin storage, and increased serotonin turnover mediated by monoamine oxidase (MAO). Persistent SARS-CoV-2 in the gut of long COVID patients is shown to cause chronic inflammation, which further reduces tryptophan absorption and, consequently, serotonin production. This depletion disrupts vagus nerve signaling, potentially leading to symptoms like memory loss commonly seen in long COVID. Such findings suggest that the virus’s continued presence in the gut, rather than in the cardiovascular system or CNS, could explain these symptoms.

Additionally, severe inflammation in COVID-19 has been linked to the exhaustion of CD4^+^ T cells, vital for transitioning from inflammation to repair during wound healing. This exhaustion might contribute to sustained inflammation, exacerbating long COVID symptoms. The interaction between the immune and nervous systems, mediated by neurotransmitters, hormones, and cytokines, plays a significant role in this process. Serotonin, in particular, is crucial in both the immune system and inflammatory responses [137,138].

Consequently, the reduction in peripheral serotonin in long COVID patients impairs vagus nerve activity and affects hippocampal responses and memory functions. This provides a plausible explanation for the neurocognitive symptoms associated with viral persistence in long COVID and possibly other post-viral syndromes. Excessive immune cell activation and inflammation, a hallmark of COVID-19, may impair various organ functions, leading to symptoms like respiratory failure, headache, impaired consciousness, and severe neurological disorders, including encephalitis [137].

In light of these findings, hyperbaric oxygen therapy (HBOT) has been suggested as a treatment option for long COVID [139]. HBOT involves breathing near 100% oxygen intermittently in a pressurized hyperbaric chamber and has shown efficacy in treating chronic fatigue syndrome [140,141]. Another proposed treatment method for long COVID is Vitamin C administration. Due to its immune-boosting properties and role in neurotransmitter production and cholesterol metabolism, Vitamin C is a promising candidate for managing COVID-19 symptoms [142,143].

### Risk Factors Contributing to the Development of Long COVID

Risk factors for severe COVID-19, leading to hospital admission and increased mortality, include advanced age, male sex, non-white ethnicity, disability, and existing comorbidities like obesity, cardiovascular disease, respiratory conditions, and hypertension [144,145]. Conversely, determinants for long COVID-19 are not as well established.

Emerging studies indicate that a severe initial phase of COVID-19 may predispose individuals to long-term sequelae [146]. This correlation is supported by the study of Sudre et al. [147], who observed that experiencing over five symptoms in the first week of illness was associated with prolonged COVID-19. They reported a tripling in the incidence of long COVID-19 among those with a severe initial infection. This association is corroborated by findings from Pływaczewska-Jakubowska and coworkers [148], who noted that long COVID-19 was significantly more prevalent in patients who experienced severe acute symptoms.

However, certain risk factors for acute COVID-19 do not necessarily predispose individuals to long COVID-19. Pazukhina et al. [149] conducted a prospective cohort study and noted a distinction between sexes: while men were more prone to acute COVID-19, women were more likely to suffer from long-term symptoms, contradicting earlier acute phase observations. They found a doubled risk of prolonged symptoms in women at both 6 and 12 months post-infection, aligning with previous research [82]. The sustained elevation of the inflammatory marker IL-6 in women and the heightened activity of T cells—attributable to the double presence of the X chromosome, which contains numerous immune-related genes—may contribute to this disparity [150,151]. Additional factors, such as stress, poor sleep quality, and depression, may exacerbate long COVID-19 in women [152].

Age is another crucial risk factor. Specific age demographics, notably individuals aged 35–49 years (26.8%), 50–69 years (26.1%), and 70 years or older (18%), are more susceptible to enduring symptoms of SARS-CoV-2 infection [153].

Chronic health conditions also influence long COVID-19 susceptibility. Pre-existing asthma, for instance, has been strongly associated with persistent COVID-19 symptoms [91,147]. Additionally, chronic inflammation and obesity-related immunometabolic disturbances may not only exacerbate the acute phase but also contribute to long COVID-19 syndrome [154]. Debski et al. [155] reported a higher BMI as a risk factor for post-COVID-19 syndrome. Interestingly, ethnicity seems to differ in its impact on long COVID-19, with non-white ethnic minority groups showing a lower risk of developing prolonged symptoms [156,157].

Notably, a comparison between the recovery outcomes in the upper airway versus alveolar inflammation highlights the complexity of COVID-19’s impact on the lungs and the importance of tailored post-recovery monitoring and care. Patients with upper airway inflammation typically experience a full recovery, with the restoration of normal oxygen transfer to the blood and healing of any vessel injury. This is mainly because the inflammation is less severe and does not typically lead to permanent lung damage [158]. In contrast, patients suffering from alveolar inflammation, which involves the deeper lung tissues and air sacs, face a more complicated recovery [56]. This type of inflammation often leads to persistent fibrosis—a condition where lung tissue becomes scarred and stiff. Such fibrosis impairs the lung’s ability to effectively transfer oxygen to the blood [159]. This ongoing impaired oxygenation can lead to a failure in healing vessel injuries, meaning that microclots formed as part of the body’s response to vessel injury might persist even after the lung inflammation has subsided [160]. These findings are evident in studies that have analyzed pulmonary function and inflammation in COVID-19 patients. Pantofli et al. [53] examined bronchoalveolar lavage (BAL) samples from COVID-19 patients, revealing significant differences in leukocyte profiles between ICU and IMW patients, which could indicate varying degrees of lung inflammation and recovery outcomes. Additionally, research on exhaled nitric oxide in patients recovering from COVID-19 has shown that residual inflammation in the distal lung (including the alveoli) can persist, particularly in those who initially had a severe form of the disease. This ongoing alveolar inflammation might contribute to the long-term development of pulmonary fibrosis, underscoring the need to regularly monitor pulmonary function and inflammation in COVID-19 patients [161].

## 4. Potential Therapies for Long COVID-19 Syndrome

Several guidelines have been developed that focus on treating and managing long COVID-19. For example, NICE has proposed comprehensive assessment, investigation, and management approaches (https://www.nice.org.uk/guidance/ng188/resources/covid19-rapid-guideline-managing-the-longterm-effects-of-covid19-pdf-51035515742; accessed on 15 October 2023). Similarly, the NIH has released treatment guidelines for COVID-19, but these offer limited guidance for managing long-term COVID-19 effects (https://www.covid19treatmentguidelines.nih.gov/, accessed on 15 October 2023).

While a significant portion of research has appropriately focused on the acute phase of COVID-19, there is a growing recognition of the need to address the long-term effects of the disease. In this context, drug repurposing is emerging as a critical area of investigation. Antihistamines are under consideration following cellular studies indicating that histamine-1 receptor antagonists might inhibit SARS-CoV-2 entry into cells expressing the ACE2 receptor, but their efficacy for treating long COVID-19 remains to be established [30,162].

Monoclonal antibodies like Leronlimab, which is used for HIV and has been shown to reduce viral plasma levels in acute COVID-19 patients, are being investigated for their potential to mitigate long-lasting COVID-19 symptoms [163]. Tocilizumab, which blocks interleukin-6 receptors, was tested in a small clinical trial for acute COVID-19, and research into its long-term effects is ongoing. Melatonin, noted for its antioxidant properties, is also being considered for treating long-term COVID-19 effects (reviewed in [164]).

For the cardiovascular manifestations of long COVID-19, NICE guidelines suggest beta-blockers as treatment options for conditions such as angina, cardiac arrhythmias, and acute coronary syndromes [165]. Sulodexide has been found to reduce symptom severity in patients with endothelial dysfunction [166]. The effectiveness of Cognitive Behavioral Therapy (CBT) has been questioned due to reported adverse effects [167]. The use of intravenous vitamin C to alleviate fatigue in long COVID-19 patients has been recently reviewed [168].

The persistent neurological complications post-COVID-19 have made Biofeedback (BFB) therapy an area of interest, with potential benefits for headaches, seizures, and insomnia, and Neurofeedback (NFB) has been documented for its long-term effectiveness [169]. For long-term neurological symptoms, glucocorticoids may be beneficial [170], and medications like tryptans and indomethacin could address prolonged symptoms such as headaches [171,172].

Pulmonary symptoms often persist post-acute COVID-19. Critical Care guidelines recommend chest imaging for early detection of pulmonary impairment and the use of corticosteroids to improve function [173]. Hyperpolarized MRI has been cited for its ability to detect gas exchange abnormalities [174]. According to Mayo Clinic recommendations, managing factors that worsen dyspnea, such as smoking cessation and avoiding pollutants, is crucial [175]. Treatment for pulmonary fibrosis should follow idiopathic pulmonary fibrosis guidelines, and anti-fibrotic therapies are considered promising options [176]. Clinical trials also evaluate the efficacy of hyperbaric oxygen therapy, montelukast, and pirfenidone for respiratory conditions associated with long COVID.

The role of COVID-19 vaccination in addressing long COVID is multifaceted and operates on three distinct levels. Firstly, the vaccines effectively prevent SARS-CoV-2 infection, thereby reducing the risk of developing long COVID. Secondly, for vaccinated individuals who contract COVID-19, the vaccines tend to lessen the severity of the disease, which may mitigate the development or intensity of long COVID symptoms. Finally, emerging evidence suggests that vaccination may also benefit those already suffering from long COVID, potentially alleviating some of the persistent symptoms associated with the condition [33,177].

A significant reduction in the incidence of long COVID following vaccination was reported in a systematic review by Byambasuren et al. [177]. In a study by Tran et al. [178], the first dose of the COVID-19 vaccine was associated with decreased severity of the disease and improved impacts on patients’ social, professional, and family lives. The study also found that vaccination led to a higher remission rate of long COVID symptoms and an increased proportion of patients reporting an acceptable symptom state. However, it is essential to note that a small percentage of patients experienced adverse effects, and some reported a worsening of symptoms or relapses post-vaccination [179].

Nayyerabadi et al. [180] also reported significant improvements in long COVID patients post-vaccination, including decreased symptoms and affected organ systems, and increased WHO-5 Well-Being Index Scores. This study suggested that vaccination helps in reducing systemic inflammation in long COVID patients. Despite these improvements, the persistence of SARS-CoV-2 S1 antigen in non-classical monocytes, regardless of vaccination status, indicates an ongoing inflammatory process.

Overall, vaccination before SARS-CoV-2 infection has been associated with reduced risks or odds of long COVID [181]. This is highlighted in a recent systematic review by Notarte et al. [182] which induced eleven studies involving 36,736 COVID-19 survivors to investigate changes in long-COVID symptoms after vaccination. While most studies showed improvement in symptoms post-vaccination, a few reported no change or worsening symptoms. The comprehensive impact of COVID-19 vaccination on long COVID continues to be an active research area.

## 5. Conclusions

To conclude, this work reviewed the association between long COVID and its implications for the respiratory, cardiovascular, and nervous systems. Numerous individuals have been, and continue to be, affected by the SARS-CoV-2 pathogen, leading to global concerns about the long-term effects of the virus. These post-acute manifestations of infection are emerging as a complex and diverse syndrome, impacting various bodily systems, including the cardiac, nervous, and respiratory systems, which this research investigates. These effects contribute to multiple symptoms that diminish the quality of life, functional capacity, and workability. Currently, the nature of long COVID-19 is elusive, with many unanswered questions. A more profound comprehension of its pathogenesis, risk factors, symptoms, and treatment are imperative. The definition of ‘long symptoms’ remains uncertain due to variations in the duration described across studies, presenting a wide array of individuals experiencing persistent symptoms for weeks or more post-infection. While data on the effects of vaccination are limited, the findings are promising, underscoring the critical need for investment in research and healthcare resources to alleviate the enduring health, social, and economic repercussions of the post-COVID-19 condition.

## Figures and Tables

**Table 1:** Examples of studies examining the long-term respiratory system symptoms attributable to SARS-CoV-2 infection.

n	Follow Up ^1^	Main Findings/Symptoms	Ref
478	4 months	16.3% (78 patients) reported new-onset dyspnea. 4.8% (23 patients) experienced new-onset cough. Patients with new-onset dyspnea: Were younger on average (56.1 years) compared to those without dyspnea (61.9 years). Had more severe COVID-19, with 56.4% requiring ICU ^2^ admission compared to 24.5% in the non-dyspnea group. Had a higher incidence of pulmonary embolism (18.0% vs. 6.8%). Among patients reassessed at ambulatory care, 19.3% had fibrotic lung lesions, with 97% showing lesions covering less than 25% of the lung. Patients with fibrotic lesions: Were older on average (61 years) compared to those without lesions (56 years). More frequently required ICU management (87.9% vs. 47.4%). Had lower total lung capacity and diffusing capacity of the lung for carbon monoxide (DLCO). A combination of new-onset dyspnea, fibrotic lesions, and DLCO below 70% predicted was observed in 8 out of 478 patients.	[34]
76	3 months	Commonly included fever, sputum production, fatigue, diarrhea, dyspnea, cough, chest tightness on exertion, and palpitations. Serum troponin-I levels during acute illness were highly correlated with post-discharge fatigue. Lymphopenia was correlated with post-discharge symptoms of chest tightness and palpitations on exertion. Lung function tests like FEV1, FVC, FEV1/FVC, total lung capacity, and diffusion capacity were mostly normal (>80% predicted). Lung HRCT scans returned to normal in 82% of patients. However, 42% of participants exhibited mild pulmonary function abnormalities 3 months post-discharge.	[37]
200	42 days	The most frequent initial symptom was dyspnea (59.5%), followed by weakness (55.5%), myalgia (53.5%), and shivering (51.5%). Six weeks post-discharge: None of the patients were readmitted. 42% (94 patients) were symptom-free. The most common lingering symptom was fatigue (19.5%), followed by dyspnea (18.5%), weakness (18%), and activity intolerance (14.5%).	[36]
119	61 days	87% of patients showed radiographic resolution of pulmonary infiltrates. 44% had modified Medical Research Council Dyspnea scale grades above their pre-COVID-19 baseline. Persistent symptoms included: Fatigue in 68%. Sleep disturbances in 57%. Breathlessness in 32%. 38% had a slow 4-m Gait Speed (4MGS) of less than 0.8 m per second. 35% showed a desaturation of >4% during the Sit-to-Stand test. Out of 56 thoracic computed tomography scans, 75% revealed COVID-19-related interstitial and/or airway disease.	[38]
41	7 months	Common CT patterns of abnormalities included: Parenchymal band in 41%. Interlobular septal thickening in 32%. Traction bronchiectasis in 29%. 61% of patients showed complete radiological resolution. 29% of patients developed pulmonary fibrosis. Oxygen consumption and metabolic equations were decreased, and the ventilatory equivalent for CO_2_ was increased in the fibrosis group. Older patients with severe conditions are more prone to fibrosis, potentially leading to cardiopulmonary insufficiency.	[39]
59	30 days	89.8% (53/59) showed a typical transition from early to advanced and then to dissipating phases of the disease. 39% (23/59) developed fibrosis (group A), while 61% (36/59) did not show definite fibrosis group B). Comparisons between the two groups showed: Group A patients were older (mean age 45.4 vs. 33.8 years). They had longer length of stay in the hospital (19.1 vs. 15.0 days). Higher rate of ICU admission (21.7% vs. 5.6%). Pulmonary fibrosis can develop early post-hospital discharge in COVID-19 patients. Older patients with severe illness are more prone to developing fibrosis, as indicated by thin-section CT results.	[40]

^1^ Median time between COVID-19 diagnosis and examination of participants post-diagnosis. ^2^ ICU: Intensive Care Unit.

**Table 2:** Examples of studies examining the long-term cardiovascular system symptoms attributable to SARS-CoV-2 infection.

n	Follow Up ^1^	Main Findings/Symptoms	**Ref**
100	71 days	Cardiovascular issues were detected in 78% of participants. Ongoing myocardial inflammation was detected in 60% of participants.	[65]
26	47 days	58% (15 patients) had abnormal findings on conventional CMR ^2^ imaging. Myocardial edema was detected in 54% (14 patients). LGE ^3^ was observed in 31% (8 patients). Patients with positive CMR results had reduced right ventricle function, including lower ejection fraction, cardiac index, and stroke volume/body surface area. Quantitative mapping showed significant increases in global native T1, T2, and extracellular volume in patients with positive CMR results.	[66]
384	54 days	In the study population: 53% reported persistent breathlessness. 34% continued to experience cough. 69% suffered from ongoing fatigue. 38% of chest radiographs (X-rays) remained abnormal. 9% of the chest radiographs showed deterioration.	[67]
148	68 days	54% of the patients had late LGE and/or ischemia. Myocarditis-like scar in 26% of patients. Myocardial infarction was found in 19% of patients. 66% of patients with ischemic injury have no prior history of coronary disease.	[68]
346	109 days	73% of participants reported cardiac symptoms at the time of measurement. Common cardiac symptoms included exertional dyspnea (62%), palpitations (28%), atypical chest pain (27%), and syncope (3%). Symptomatic individuals showed higher heart rates and increased imaging markers or contrast agent accumulation, suggesting cardiac inflammation 329 days post-infection: 57% still experienced cardiac symptoms.	[69]
180	≥12 weeks	In the study population: 52% reported shortness of breath or fatigue. 34% experienced chest pain or discomfort. 37% had heart palpitations or arrhythmias. Acute pericarditis was diagnosed in 39 patients. Mild-to-moderate pericardial effusion was found in 12 patients. 27 patients had thickened and bright pericardial layers with small effusions (<5 mm) and possibly pericardial B-lines. Heart palpitations/arrhythmias and autoimmune/allergic disorders were significantly associated with acute pericarditis.	[47]

^1^ Median time between COVID-19 diagnosis and examination of participants post-diagnosis. ^2^ CMR: Cardiac Magnetic Resonance. ^3^ LGE: Late gadolinium enhancement.

**Table 3:** Examples of studies examining the long-term symptoms of SARS-CoV-2 infection in the nervous system.

n	Follow Up ^1^	Main Findings/Symptoms	Ref
1276	6 months–1 Year	Overall sequelae symptoms decreased from 68% at 6 months to 49% at 12 months. Incidence of dyspnea increased slightly from 26% to 30% between the 6-month and 12-month visits. Anxiety or depression increased from 23% at 6 months to 26% at 12 months. No significant change was observed in the 6-min walk distance between the two visits. 88% of previously employed patients returned to their original work by 12 months. Women had higher odds than men of experiencing fatigue or muscle weakness, anxiety or depression, and diffusion impairment (related to lung function). At 12 months, participants had more mobility issues, pain, discomfort, and anxiety or depression compared to controls and exhibited more prevalent symptoms.	[82]
2433	1 year	Common symptoms included fatigue, sweating, chest tightness, anxiety, and myalgia. Higher fatigue risk was associated with older age, female sex, and severe disease during hospital stay. Older age and severe disease increased the likelihood of having at least three symptoms. Median CAT (COPD Assessment Test) score was 2, with 6.6% (161 patients) having a score of at least 10. Severe disease and coexisting cerebrovascular diseases were independent risk factors for a CAT score of at least 10.	[89]
165	6 months	Patients experienced a range of symptoms: Fatigue was reported by 34%. Memory or attention issues by 31%. Sleep disorders by 30%. 40% of patients showed neurological abnormalities, including: Hyposmia (loss of smell) in 18.0%. Cognitive deficits in 17.5%. Postural tremor in 13.8%. Subtle motor or sensory deficits in 7.6%. Factors such as older age, pre-existing comorbidities, and the severity of COVID-19 were independent predictors of neurological issues. Pre-existing vulnerabilities and the severity of SARS-CoV-2 infection influence the prevalence and severity of long-term neurological manifestations	[90]
120	110 Days	Persistent symptoms among the participants included: Fatigue in 55%. Dyspnea in 42%. Memory loss in 34%. Concentration and sleep disorders in 28% and 30.8%, respectively. No significant differences in symptoms were found between patients treated in general wards and those in ICU ^2^. Health-related quality of life aspects like mobility, self-care, pain, anxiety or depression, and usual activities were affected in both groups, with a slight difference in pain in the ICU group.	[84]
100 (32 in ICU, 62 in the hospital ward)	48 Days	New, illness-related fatigue was reported by 72% of participants in the ICU group and 60.3% in the ward group. Breathlessness was the second most common symptom, vis., 65.6% in the ICU group and 42.6% in the ward group. Psychological distress: 46.9% in the ICU group and 23.5% in the ward group. A clinically significant drop in EQ5D (a measure of health-related quality of life) was observed in 68.8% of the ICU and 45.6% of the ward groups.	[91]
236, 379	6 Months	Within 6 months post-diagnosis, 33.62% had a neurological or psychiatric diagnosis, while 12.84% received their first diagnosis. For patients admitted to Intensive Therapy Units (ITUs): 46.42% received a neurological or psychiatric diagnosis, and 25.79% had their first diagnosis. Specific incidence rates in the whole COVID-19 cohort included: Intracranial hemorrhage: 0.56%.; Ischemic stroke: 2.10%; Parkinsonism: 0.11%.; Dementia: 0.67%; anxiety disorder: 17.39%; Psychotic disorder: 1.40%. In the ITU group, the above incidences were higher: Intracranial hemorrhage: 2.66%; Ischemic stroke: 6.92%; Parkinsonism: 0.26%; Dementia: 1.74%; Anxiety disorder: 19.15%; Psychotic disorder: 2.77%. Neurological and psychiatric diagnoses were more common in COVID-19 patients than those with influenza or other respiratory tract infections.	[92]

^1^ Median time between COVID-19 diagnosis and examination of participants post-diagnosis. ^2^ ICU: Intensive Care Unit.

## Data Availability

No new data were created in this study.
